# Galectin-12 in the Regulation of Sebocyte Proliferation, Lipid Metabolism, and Immune Responses

**DOI:** 10.3390/biom15060837

**Published:** 2025-06-08

**Authors:** Ching-Han Tsao, Wei-Chen Hsieh, Feng-Jen Lin, Fu-Tong Liu, Ri-Yao Yang

**Affiliations:** 1 Department of Dermatology, Keck School of Medicine, University of Southern California, Los Angeles, CA 90089, USA; chinghan.tsao@med.usc.edu; 2 AHMC Dermatology and Research Center, Arcadia, CA 91007, USA; sophia.hsieh@ahmcdnr.com; 3 Institute of Biomedical Sciences, Academia Sinica, Taipei 115, Taiwan; fongrenlin@gate.sinica.edu.tw

**Keywords:** galectin-12, adipose tissue, sebaceous glands, PPARγ

## Abstract

Galectin-12, a member of the galectin family of glycan-binding proteins, exhibits restricted tissue distribution, primarily in adipocytes and sebocytes. In sebocytes, it modulates the cell cycle, influences lipid metabolism through interactions with peroxisome proliferator-activated receptor γ (PPARγ), and exerts immunomodulatory functions by activating immune signaling pathways. Dysregulation of sebocyte homeostasis, lipid metabolism, and immune responses contributes to the pathogenesis of a number of skin diseases, underscoring the therapeutic potential of targeting galectin-12. The review summarizes and discusses current developments in the field to foster future research in this important but underexplored galectin.

## 1. Introduction

Sebocytes, the main cellular component of sebaceous glands (skin appendages), are akin to adipocytes in that they are mainly composed of fat and are essential for maintaining skin homeostasis. This review focuses on the functions of galectin-12, a member of the galectin family of glycan-binding proteins, initially discovered in adipocytes and subsequently found in sebocytes, in adipocyte differentiation and lipid metabolism, as well as in sebocyte proliferation, lipogenesis, and immune responses.

Galectins are a family of β-galactoside-binding lectins characterized by one or two conserved carbohydrate recognition domains (CRDs), each approximately 130 amino acids long [1]. The galectin family consists of over 16 members, classified into three structural types: prototype, chimera, and tandem-repeat galectins [2]. Prototype galectins (e.g., galectin-1, -2, -5, -7, -10, -11, -13, -14, -15, and -16) contain a single carbohydrate recognition domain (CRD) and can form homodimers [3]. Galectin-3, the only chimera-type member, has a C-terminal CRD and an N-terminal domain consisting of proline- and alanine-rich tandem repeats [3]. Tandem-repeat galectins, including galectin-4, -6, -8, -9, and -12, contain two distinct CRDs connected by a short peptide linker [3]. The N-terminal CRD is more similar to other galectin CRDs than the C-terminal one.

Galectins are primarily recognized for their ability to bind β-galactosides, and they exhibit specific binding preferences for various oligosaccharides [4,5]. For instance, galectin-1 and galectin-7 show distinct affinities for type I and type II lactose (Lac)/N-acetyl lactosamine (LacNAc) residues and can recognize fucosylated neutral glycans [6]. In contrast, galectin-3 has a high affinity for poly-LacNAc structures, such as *Lacto-N-neohexaose* (LNnH) and *Lacto-N-neooctaose* (LNnO), highlighting its role in recognizing complex glycan structures [6,7]. Notably, galectin-12, a tandem-repeat type, preferentially binds 3-fucosylated lactose derivatives, such as Lewis X (LeX) [8]. Although its three-dimensional structure remains unresolved, computational models indicate that galectin-12 has a highly hydrophobic structure with five distinct hydrophobic zones, as determined through in silico sequence analysis [8].

Galectins are expressed in a variety of tissues, either constitutively or in an inducible fashion, with family members exhibiting different tissue expression patterns [1]. They participate in various physiological and pathological processes and can function extracellularly or intracellularly. While many studies have explored extracellular functions using exogenously added galectins to cultured cells, these may not reflect the functions of endogenous galectins [9,10]. On the other hand, intracellular galectins have been shown to regulate diverse processes through protein–protein and protein–glycan interactions [2,10]. The functions described for galectin-12 that are summarized below are all intracellular ones.

Among this family, galectin-12 remains underexplored, partly due to technical difficulties (as highlighted in the Section 4). The review focuses on galectin-12 for its uniqueness among galectins in localization, cell-type specificity, and function. We hope to provide a perspective to draw more attention to the field to catalyze innovative studies of this important protein.

Galectin-12 was found by sequencing a clone from a cDNA library of human Jurkat T-cells synchronized at the G1 phase of the cell cycle. The results showed sequence homologies to galectins, suggesting it could be a cDNA for a new member of the galectin family [11]. The authors cloned the full-length cDNA and several alternatively spliced variants from the human retina and the myeloid leukemia cell line HL-60 using 5′-RACE (Rapid Amplification of cDNA Ends) [11]. Based on the cDNA sequence, they located the galectin-12 gene on human chromosome 11q13 and showed that its exon organization closely resembles that of other galectin genes. The galectin-12 protein consists of two carbohydrate-recognition domains (CRDs) connected by a linker sequence.

The N-terminal CRD has moderate similarity (32–43% sequence identity) to most other galectin CRDs, while the C-terminal CRD exhibits significant divergence (23–28% sequence identity) [12]. Several features of the cDNA, including a suboptimal sequence context for translation initiation and AT-rich elements in its 3′-untranslated region [11], are commonly found in those for proteins with growth regulatory functions [11,13,14]. Indeed, Yang et al. found that galectin-12 gene expression is significantly upregulated when cells are synchronized at the G1 phase or the G1/S boundary. When the protein was ectopically expressed in the human cervical cancer cell line HeLa, it suppressed cell proliferation by arresting cell-cycle progression at the G1 phase [11]. At that time, the researchers were not able to detect the galectin-12 protein. Research was then focused on galectin-12 in adipocytes after it was found to be preferentially expressed in mouse and human adipocytes and to regulate adipogenesis and lipid metabolism [15,16,17]. Subsequently, galectin-12 was also examined in porcine adipocytes, revealing distinct regulatory mechanisms in intramuscular (IM) and subcutaneous (SC) fat [18]. Knockdown of the galectin-12 gene resulted in 1075 and 3016 differentially expressed genes (DEGs) in IM and SC adipocytes, respectively [19]. Pathway analysis identified PI3K-AKT and fatty acid metabolism in IM adipocytes, while TNF and WNT signaling dominated in SC adipocytes, linking galectin-12 to fat deposition and pork quality [19]. It was thus concluded that galectin-12 knockdown inhibited adipogenesis via the PKA-Erk1/2 pathway, reducing lipid droplets, downregulating adipogenic markers, and promoting lipolysis [18].

Yang et al. set out to identify galectin-12 binding protein in an attempt to elucidate galectin-12’s mechanism of action. Through the retroviral expression of FLAG-tagged galectin-12 in adipocytes, followed by immunoprecipitation using an anti-FLAG antibody to isolate protein complexes containing galectin-12, and proteomic analysis, VPS13C, a mammalian ortholog of the yeast vacuolar protein sorting 13 (Vps13), was identified as a galectin-12-binding protein [20]. VPS genes were identified in yeast as those whose deficiency leads to defects in the sorting of vacuolar (lysosomal) proteins. Various important cellular functions have been identified for several of these genes and their mammalian orthologs [21,22,23]; however, less is known about VPS13, primarily due to the substantial size of the gene and its corresponding protein. The VPS13C gene, similar to galectin-12, is upregulated during adipocyte differentiation, and the two proteins co-localize in lipid droplets [20]. Using a retro-lentiviral system for conditional gene knockdown, the researchers discovered that the protein is essential for both the stability of galectin-12 and adipogenesis [20]. Recent studies conducted by various researchers have identified VPS13C as a lipid transport protein located at the contact sites between the endoplasmic reticulum (ER) and lipid droplets, lysosomes, and mitochondria [24]. This protein plays a crucial role in regulating the dynamics and functions of these organelles [24,25,26,27,28]. Importantly, loss-of-function mutations in VPS13C are associated with a form of early-onset Parkinsonism with a distinct phenotype of severe and rapid progression [27].

Beyond its established role in lipid droplets, galectin-12 has also been implicated in broader metabolic networks within cancer cells. Ten novel galectin-12 interactors were identified in colon cancer cells, including the amino acid transporter SLC1A5. Galectin-12 restricts glutamine anaplerosis by reducing uptake and impacting biomass synthesis, though cancer cells compensate under low-glutamine conditions [29]. These findings raise the possibility that galectin-12 may interact with a wider range of intracellular partners, suggesting additional functional contexts beyond those previously characterized. However, the physiological or pathological implications of these interactions are less clear, as they were identified in cell lines that do not express endogenous galectin-12, but were transfected to ectopically express the protein.

## 2. Role of Galectin-12 in Sebaceous Glands

Sebaceous glands are exocrine structures associated with hair follicles [30] and are widely distributed across the skin of most mammals, including humans and rodents [31]. These glands are primarily composed of sebocytes, which are the main cell type responsible for the synthesis and secretion of sebum. Sebum, a complex mixture of lipids and cellular debris, is secreted through a holocrine mechanism in which the entire sebocyte undergoes apoptosis to release its contents. The composition of sebum is species-specific, reflecting the distinct physiological requirements of the skin in different organisms. In humans, the primary components of sebum consist of triglycerides and free fatty acids, wax esters, squalene, and cholesterol in various proportions [32].

Sebum constitutes approximately 90% of the lipids found on the skin’s surface [31]. These lipids play vital roles in preserving skin hydration, defending against environmental stressors, and preventing microbial infiltration, including bacterial invasion [33,34,35]. Sebum production is tightly linked to sebocyte differentiation, during which basal sebocytes mature into lipid-producing cells, leading to lipid droplet accumulation [36,37]. Lipid synthesis—including fatty acids, triglycerides, and cholesterol—is essential for sebum production. The production and secretion of sebum are tightly regulated to maintain skin barrier integrity. Disruptions in this regulation have been linked to various skin disorders [38,39,40].

***Peroxisome proliferator-activated receptors (PPARs).*** Among the various intracellular regulators in sebocytes, PPARs are particularly relevant to this review because of their close association with several functions of galectin-12, which will be discussed below. PPARs are nuclear receptors that regulate gene expression in response to specific ligands, including fatty acids and other lipids. Among the three PPAR isoforms—PPARα, PPARβ/δ, and PPARγ [41,42]—PPARγ plays a particularly critical role in sebocyte function by regulating processes such as sebocyte proliferation and lipid synthesis, both of which are essential for sebum production [43,44,45]. PPARγ regulates the expression of genes involved in lipid metabolism, including DGAT1 (diacylglycerol O-acyltransferase 1), an enzyme that catalyzes the final step in triglyceride synthesis and is essential for triglyceride storage within sebocytes [46]. Additionally, PPARγ regulates the expression of ACS2 (acetyl-coenzyme A synthetase 2), an enzyme that plays a crucial role in the activation of fatty acids and the synthesis of triglycerides and cholesterol—essential components of sebum [47]. This regulation ensures proper lipid production, thereby maintaining the skin’s barrier function and hydration [48].

***Galectin-12 Regulates Sebocyte Proliferation.*** Previous studies have demonstrated the expression of various hormone receptors in the pilosebaceous unit: (1) sebocytes contain 5-alpha-reductase, which converts relatively weak circulating androgens, such as dehydroepiandrosterone sulfate, into more potent androgens like dihydrotestosterone (DHT); (2) topical treatment with the androgen receptor (AR) antagonist RU58841 or 5α-reductase inhibitors (finasteride and MK386) on the dorsal skin of male fuzzy rats resulted in the inhibition of sebocyte proliferation, sebum production, and sebaceous gland size; (3) the activity of 5-alpha-reductase has been shown to be higher in the scalp and facial skin compared to non-acne-prone areas; and (4) the regulation of sebocyte proliferation and sebum production has been implicated in the development of sebaceous gland disorders such as acne vulgaris, sebaceous gland hyperplasia, sebaceous gland carcinoma, hair follicle nevus, and primary cicatricial alopecia [49,50,51,52,53,54]. In contrast, sebaceous glands are often reduced in size or absent in conditions such as psoriatic plaques, hidradenitis suppurativa lesions, linear morphea, and primary cicatricial alopecia [55,56,57]. Understanding the mechanisms that regulate the size and proliferation of sebaceous glands is essential for developing therapies for skin disorders associated with sebaceous gland dysfunction.

The role of galectin-12 in regulating sebocyte proliferation and cell-cycle progression was investigated [58]. The authors discovered that galectin-12 is highly expressed in sebocytes, and its expression is regulated during cellular proliferation. Galectin-12 positively influences sebocyte proliferation, as demonstrated by a decrease in cell proliferation in SZ95 human sebocytes when galectin-12 expression was downregulated. Similarly, galectin-12 KO mice exhibited significantly reduced sebocyte proliferation, as evidenced by a lower number of Ki-67-positive cells and smaller sebaceous glands. These results suggest that galectin-12 plays a crucial role in promoting sebocyte growth and maintaining the size of sebaceous glands.

Galectin-12 was shown to regulate cell-cycle progression by modulating critical regulators, particularly cyclin A1 and CDK2 [58]. These proteins are essential for the transition from the G1 phase to the S phase of the cell cycle, and their proper regulation is crucial for normal cell proliferation [59](. Galectin-12 was shown to upregulate the expression of cyclin A1 and CDK2, facilitating the progression of sebocytes through the cell cycle and into the S phase, thereby promoting sustained proliferation [58]. In contrast, the depletion of galectin-12 via siRNA resulted in decreased levels of cyclin A1 and CDK2, leading to cell-cycle arrest in the G1 phase and inhibiting sebocyte proliferation. This underscores the critical role of galectin-12 in regulating the cell cycle and promoting the growth of sebocytes.

The study also demonstrated that the overexpression of galectin-12 accelerated cell-cycle progression by increasing the percentage of cells in the G2/M phase, thereby further supporting its role as a key regulator of sebocyte proliferation. However, the exact mechanism by which galectin-12 regulates the expression of these cell-cycle proteins remains unclear and requires further investigation.

Beyond its role in cell-cycle regulation, galectin-12 has also been found to participate in androgen-stimulated sebocyte proliferation [58]. Androgens, such as testosterone and 5α-dihydrotestosterone (5α-DHT), are known to promote the proliferation of sebocytes. The study demonstrated that the downregulation of galectin-12 in SZ95 sebocytes inhibited androgen-induced cell proliferation. Moreover, in male galectin-12 KO mice, testosterone treatment did not lead to the enlargement of sebaceous glands, underscoring the significance of galectin-12 in mediating androgenic effects on sebocyte growth [58].

The relationship between galectin-12 and PPARγ was also investigated in this study. PPARγ plays a crucial role in cell proliferation, differentiation, and lipogenesis. The dysregulation of PPARγ signaling is associated with various pathologies, including tumors in tissues such as the breast, prostate, and colon. PPARγ expression was found to be reduced in galectin-12 knockdown SZ95 sebocytes and in galectin-12 KO mouse skin, further implicating galectin-12 in the regulation of cell-cycle progression and sebocyte proliferation [58]. This suggests that galectin-12 may interact with multiple signaling pathways, including androgen receptors and PPARγ, to regulate sebocyte function.

***Galectin-12 Modulates Sebocyte Differentiation and Lipid Metabolism.*** As mentioned above, sebaceous glands are responsible for producing sebum, a lipid-rich substance that plays a crucial role in skin hydration and protection; dysregulated lipid production in these glands can lead to skin disorders [32,60,61].

The mechanisms by which galectin-12 influences sebocyte differentiation and lipid production have been elucidated in a recent study [62]. As sebocytes differentiate, they accumulate lipid droplets, which are characteristic of both sebocyte differentiation and increased lipid secretion. Galectin-12 expression is upregulated during this process, and its presence is essential for the proper expression of key sebocyte markers, such as keratin 7 (KRT7), melanocortin-5 receptor (MC5R), and PRDM1/BLIMP1, which are indicative of both basal and mature sebocyte phenotypes [62]. In addition, galectin-12 overexpression in the human keratinocyte cell line HaCaT was shown to enhance sebocyte differentiation, suggesting that it acts as a positive regulator of this process [62].

In addition to its role in differentiation, galectin-12 also modulates lipid biosynthesis in sebaceous glands [62]. The study identifies two critical enzymes involved in lipid metabolism: DGAT1 and ACS2, which play essential roles in the formation of triglycerides and cholesterol esters [62]. The study also highlights the interaction between galectin-12 and PPARγ signaling, which is a well-known regulator of lipid metabolism [62]. In galectin-12 knockdown sebocytes, the expression of PPARγ and its downstream target genes, DGAT1 and ACS2, exhibited reduced responsiveness to linoleic acid (LA), an omega-6 fatty acid known to stimulate sebocyte differentiation and lipid production. This finding underscores the significance of galectin-12 in modulating lipid metabolism through the PPARγ pathway, further affirming its role in promoting lipogenesis.

In vivo studies with galectin-12 KO mice corroborate the in vitro findings. Although sebaceous glands produce over 90% of skin surface lipids, subcutaneous fat also contributes. To exclude the effect from subcutaneous fat, researchers used Cre/lox-dependent conditional-knockout mice in which Lgals12 was selectively deleted from sebaceous glands. Lipid samples were collected from the hair shafts of both KO and wild-type mice and analyzed using ultraperformance convergence chromatography–tandem mass spectrometry combined with gas chromatography time-of-flight mass spectrometry (GC-TOF/MS) to examine lipid composition differences. The lipid analysis demonstrates that the absence of galectin-12 leads to a significant reduction in key lipid metabolites, including cholesteryl esters, triglycerides, free fatty acids, and cholesterol. These mice exhibited lower levels of cholesteryl oleate, triglycerides, and other lipids in the sebaceous glands compared to wild-type controls, reinforcing the idea that galectin-12 is essential for normal sebaceous lipid production. These findings highlight the critical role of galectin-12 in modulating lipid homeostasis in sebaceous glands and suggest that galectin-12 deficiency can result in altered lipid composition in the skin, potentially contributing to the pathogenesis of various skin disorders.

## 3. Role of Galectin-12 in Sebaceous Gland Immune Responses

***Galectin-12 Modulates the Inflammatory Response.*** The expression and secretion of galectin-12 were explored in neutrophilic differentiation using HL-60 cells, a model for acute myeloid leukemia [63]. The study demonstrated that galectin-12 expression varied between the two neutrophil-like phenotypes induced by all-trans retinoic acid and dimethyl sulfoxide, highlighting its role in neutrophilic plasticity. Furthermore, galectin-12 expression was inhibited upon differentiation, suggesting its involvement in lipid droplet biogenesis and metabolic regulation. These findings are consistent with an early study that found high galectin-12 expression in primary human neutrophils [11], and indicate that galectin-12 could serve as a marker for neutrophilic differentiation and polarization.

Galectin-12 expression in placental tissue from women with gestational diabetes mellitus (GDM) was significantly elevated in the nucleus of syncytiotrophoblasts and extravillous trophoblasts, linking it to inflammatory processes and GDM-related complications [64]. These findings support the role of galectin-12 in metabolic inflammation and suggest that the protein may serve as a potential marker for GDM-related complications.

Galectin-12 plays multifaceted roles in metabolic inflammation. In the liver, it regulates the polarization of Kupffer cells; galectin-12 deficiency promotes M2 macrophage polarization, leading to increased TGF-β1 secretion and liver fibrosis, thereby contributing to the progression of nonalcoholic fatty liver disease (NAFLD) [65]. In the skin, plasma galectin-12 levels are elevated in psoriatic patients and correlate with metabolic indicators such as glomerular filtration rate and fasting glucose, supporting its role as a biomarker for metabolic dysfunction [66]. Moreover, galectin-12 preferentially binds 3-fucosylated glycans and modulates endothelial function and angiogenesis in adipose tissue; its expression is hypoxia-inducible and its deficiency impairs vascular network formation [8]. Collectively, these findings underscore galectin-12’s potential as a therapeutic target and biomarker in diverse inflammatory and metabolic disorders.

***Galectin-12 Modulates the Immune Response of Sebocytes.*** Sebaceous glands express pattern recognition receptors (PRRs) such as TLR2, TLR4, and the NLRP3 inflammasome, which mediate Th1 immune responses associated with conditions such as acne [45]. These receptors are activated by microbial components, such as *Cutibacterium acnes* and lipopolysaccharides, which trigger the release of cytokines and other inflammatory mediators [67,68]. This process highlights the active role of sebocytes in modulating local immune responses within the skin.

Additionally, recent data suggest that these glands modulate Th2 responses through IL-4 receptors [69]. SiRNA-mediated silencing of galectin-12 led to the suppression of both PPARγ and CCL26 expression [69], with effects similar to those produced by PPARγ antagonism (GW9662) [70]. This suggests that galectin-12 is involved in activating the PPARγ pathway, which plays a crucial role in regulating immune responses in the skin [69]. In galectin-12 conditional-KO mice (K5-Cre^+^Lgals12^fl/fl^) that are deficient in this galectin-12 in sebaceous glands, but not subcutaneous adipose tissue, the sebaceous glands exhibited reduced size and a decreased expression of PPARγ compared to the control group [69].

Moreover, galectin-12 KO mice demonstrated reduced skin inflammation in a mouse model of atopic dermatitis induced by repeated epicutaneous ovalbumin (OVA) application, as evidenced by a decrease in epidermal thickening and eosinophil infiltration [69]. The results suggest that galectin-12 contributes to the Th2 inflammatory skin response mediated by sebaceous glands. A possibility exists that galectin-12 contributes to the inflammatory response by regulating skin barrier functions and influencing lipid production from these glands. However, since total knockout rather than conditional-knockout mice were used for the study, the contribution of galectin-12 to this response through adipose tissues cannot be excluded.

RNA-seq analysis of SZ95 human sebocytes with silenced galectin-12 revealed an upregulation of ER stress-signaling proteins, including ATF and p-JNK [69], which are key regulators in the IRE1α and PERK pathways. These findings suggest that galectin-12 may regulate immune responses by modulating endoplasmic reticulum (ER) stress pathways, which are known to drive inflammation and contribute to the pathogenesis of inflammatory diseases [71].

PPARγ plays a central role in balancing Th1 and Th2 immune responses, functioning as both an anti-inflammatory regulator and a Th2 enhancer, depending on the cellular context [72]. In sebocytes, PPARγ is induced by IL-4 through the activation of STAT6, establishing a positive feedback loop that amplifies Th2-driven inflammation [69]. Recent studies have shown that PPARγ can function independently of ligand binding to facilitate transcriptional memory through chromatin remodeling, recruiting the coactivator P300 and the architectural protein RAD21 to establish a permissive chromatin state [73]. Mechanistically, this process enhances the binding of STAT6 and RNA polymerase II upon re-stimulation with IL-4, resulting in the sustained expression of Th2-associated genes [73]. Thus, galectin-12 may modulate the upregulation of PPARγ induced by IL-4 by stabilizing the chromatin environment. This stabilization could enhance PPARγ’s ability to recruit coactivators and architectural proteins, thereby influencing macrophage polarization and related immune responses [73]. Given that both galectin-12 knockdown and PPARγ inhibition (GW9662) lead to reduced CCL26 expression and improved atopic dermatitis symptoms, as described above, galectin-12 might sustain Th2 immune responses by reinforcing PPARγ activation in sebocytes [69].

The endoplasmic reticulum (ER) is a crucial regulator of cellular homeostasis, especially in lipid metabolism and immune signaling [74]. Moreover, recent studies have demonstrated that ER stress negatively regulates IL-4 signaling through the IRE1α and PERK pathways, thereby suppressing IL-4-induced transcriptional programs [69]. These pathways function as a negative feedback mechanism to prevent excessive Th2-associated inflammation [69].

Given that ER stress has been shown to repress PPARγ transcription through IRE1α-mediated suppression [75], there is a possibility that galectin-12 plays a role in sustaining PPARγ expression by preventing the activation of ER stress. Notably, the knockdown of galectin-12 did not alter the expression levels of STAT6, indicating that its regulatory effect on IL-4 responses occurs through the modulation of endoplasmic reticulum (ER) stress modulation rather than through direct interference with STAT6 activation [69]. Thus, galectin-12 may enhance IL-4 responses by directly influencing PPARγ expression and alleviating the suppression of Th2 immune signaling mediated by endoplasmic reticulum (ER) stress [69].

An intriguing aspect of galectin-12′s function is its interaction with VPS13C, a lipid transporter recognized as a binding protein for galectin-12 [20]. VPS13C is known to facilitate lipid transfer between the ER and lipid droplets, with its N-terminal domain serving as a lipid transport module [24]. Given that IRE1α and PERK are sensitive to lipid saturation levels in the endoplasmic reticulum (ER), it is plausible that galectin-12 plays a role in regulating lipid homeostasis within the ER. This regulation may affect IL-4-induced immune signaling pathways, thereby linking lipid metabolism to immune modulation in the skin.

## 4. Conclusions

**Figure 1:**
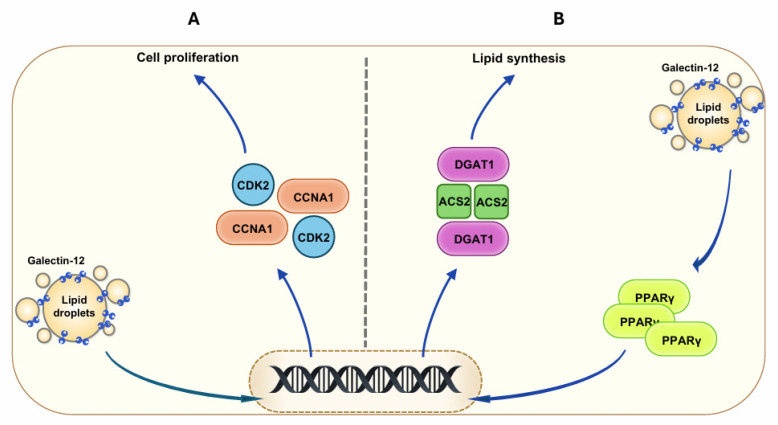
Galectin-12 regulates the proliferation and differentiation of sebocytes. (**A**) Galectin-12 promotes sebocyte proliferation by upregulating key cell-cycle regulators, including CCNA1 and CDK2. (**B**) Galectin-12 promotes sebocyte differentiation and lipogenesis by upregulating PPARγ, which drives the expression of downstream targets, including ACS2 and DGAT1, that are essential for triglyceride and cholesterol synthesis.

Galectin-12 is expressed in sebocytes and plays diverse roles in these cells, including cell-cycle progression, cell differentiation, lipogenesis, lipolysis, and inflammation. It is localized on lipid droplets and functions as a lipogenic factor. Additionally, it plays a crucial role in lipid metabolism by modulating PPARγ and its downstream targets essential for the synthesis of triglycerides and cholesterol, and by regulating lipase activity to restrict lipolysis (Figure 1). Galectin-12′s interaction with VPS13C, a lipid transport protein, implicates this protein in inter-organellar lipid transfer.

**Figure 2:**
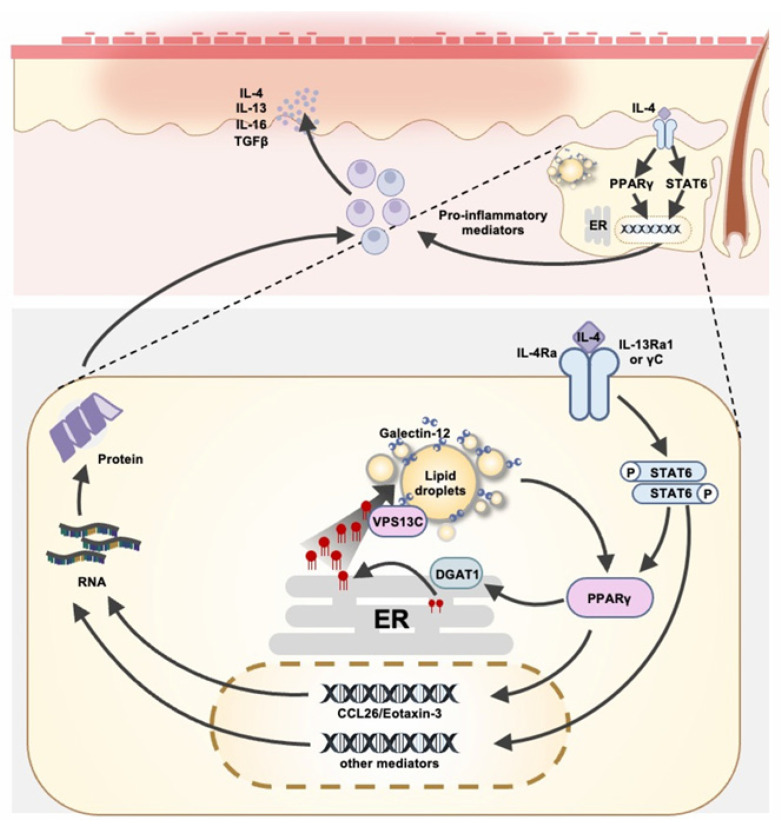
Galectin-12 contributes to the immune responses of sebocytes. Sebaceous glands contribute to cutaneous immune modulation. Lin et al. [69] demonstrated that sebocytes express IL-4 receptors and respond to IL-4 by activating STAT6 signaling and releasing CCL26, indicating their potential to participate in Th2-type responses. IL-4 stimulation also enhances PPARγ expression in both cultured sebocytes and sebaceous glands. Galectin-12 may further influence immune responses by interacting with the lipid transporter VPS13C, affecting ER stress and lipid homeostasis.

Through various mechanisms, galectin-12 plays important roles in diverse inflammatory responses, influencing neutrophilic differentiation, metabolic inflammation, and immune cell polarization. Its involvement in diseases such as gestational diabetes mellitus (GDM), non-alcoholic fatty liver disease (NAFLD), psoriasis, and atopic dermatitis suggests its potential as a biomarker for metabolic and inflammatory disorders. Galectin-12 can also modulate immune and inflammatory responses through sebocytes. In particular, galectin-12 appears to regulate immune responses in the skin by influencing sebaceous gland function, lipid metabolism, and inflammatory pathways. By modulating PPARγ, ER stress, and lipid homeostasis, galectin-12 may play a significant role in maintaining immune balance in the skin. Moreover, galectin-12 plays a crucial role in modulation of the Th2 immune response induced by key mediators such as interleukin-4 (Figure 2). This function further highlights its multifaceted contribution to maintaining skin homeostasis and overall skin health. 

Despite these significant findings, the precise molecular mechanisms by which galectin-12 regulates gene expression related to proliferation, differentiation, and immune responses have yet to be fully elucidated. The discovery of VPS13C, a galectin-12-binding protein, offers promising insights into how galectin-12 may regulate lipid transport from the ER to lipid droplets. Consistent with this, in galectin-12 knockdown models, ER stress and lipid overload were observed, further supporting the notion that galectin-12 is essential for maintaining lipid homeostasis in sebocytes.

Sebaceous gland dysfunction is involved in the pathogenesis and progression of a number of skin disorders, including acne vulgaris, atopic dermatitis, psoriasis, rosacea, seborrheic dermatitis, and certain types of alopecia [45,76]. Given the central role of galectin-12 in sebaceous gland function, galectin-12 is emerging as a promising therapeutic target. As the protein is preferentially expressed in adipocytes and sebocytes, drugs targeting this protein are unlikely to affect the function of other cell types and thus may have fewer side effects. However, further research is needed to fully understand its molecular mechanisms and to validate its therapeutic potential in managing conditions related to sebaceous gland dysfunction, particularly those associated with abnormal lipid metabolism and inflammation.

While significant progress has been made in galectin-12 studies, the field is still challenged by technical difficulties associated with the physicochemical and physiological properties of the protein. The inherent tendency of the galectin-12 protein to aggregate in vitro and its low antigenicity severely limit the availability of active soluble recombinant protein and validated antibodies for biochemical characterization and functional studies. As with other galectins, extracellular versus intracellular galectin-12 functions await further clarification. Although some studies hinted at the secretion of galectin-12, the protein clearly functions intracellularly, with most mechanisms identified for its functions involving intracellular signaling pathways, and its interactors identified (such as VPS13C) are intracellular proteins. In this regard, the use of cell-permeable and impermeable galectin inhibitors along with neutralizing antibodies in functional assays is expected to provide invaluable insights.

Given the central role of galectin-12 in sebaceous gland function and its potential involvement in skin disorders such as acne, seborrheic dermatitis, and sebaceous gland hyperplasia, galectin-12 is emerging as a promising therapeutic target. As the protein is preferentially expressed in adipocytes and sebocytes, drugs targeting this protein are unlikely to affect the function of other cell types and thus may have fewer side effects. However, further research is needed to fully understand its molecular mechanisms and to validate its therapeutic potential in managing conditions related to sebaceous gland dysfunction, particularly those associated with abnormal lipid metabolism and inflammation.

## Data Availability

Not applicable.
